# A study of the mediating effect of self-perceived burden between meaning in life and dignity in disabled patients based on a dignity theory model

**DOI:** 10.3389/fpsyg.2025.1519537

**Published:** 2025-01-27

**Authors:** Tang Rong, Ding Xing, Cheng Xianzong, Liu Ruian

**Affiliations:** ^1^School of Nursing, Chengdu Medical College, Chengdu, China; ^2^School of Public Health, Chengdu Medical College, Chengdu, China

**Keywords:** disability, self-perceived burden, meaning in life, dignity, mediating effect

## Abstract

**Objective:**

To explore the mediating role of self-perceived burden between meaning in life and the level of dignity in patients with disability.

**Methods:**

From July to October 2023, 229 disabled patients in a tertiary hospital in Sichuan Province, China were selected and surveyed using the Self-Perceived Burden Scale, Meaning in Life Questionnaire, and Patient Dignity Inventory.

**Results:**

The levels of self-perceived burden, meaning of life, and dignity of the disabled patients were 23.55 ± 9.83, 46.27 ± 9.71, and 43.38 ± 22.04 points, respectively. Self-perceived burden was positively correlated with the level of dignity (*r* = 0.460, *p* < 0.001), and meaning in life was negatively correlated with self-perceived burden and the level of dignity (*r* = −0.325, *r* = −0.526, both *p* < 0.001). The results of structural equation modeling showed that the total effect of the meaning in life of disabled patients on the level of dignity was −1.1945, the direct effect was −0.9559, and the indirect effect was −0.2386, with the indirect effect accounting for 19.98% of the total effect.

**Conclusion:**

Self-perceived burden partially mediated the relationship between the meaning of life and the dignity of patients with incapacitation. Caregivers should pay attention to the level of patients’ self-perceived burden and actively cooperate with treatment and control of disease development as the current goal of life to enhance patients’ meaning of life and improve the loss of dignity.

## 1 Introduction

The Institute of Medicine states that patient-centered care is the practice of caring for patients by respecting their individual preferences, needs, and values and ensuring that patient values guide all clinical decisions (ABIM Foundation and ACP-ASIM Foundation, 2002). Valuing dignity is no longer a basic human right but one of the most important values for health care organizations (Martin-Ferreres et al., 2019).

Due to the loss or limitation of their daily living ability, disabled patients often need to rely on others, and at the same time, due to a lack of social roles and a decrease in social acceptance, they are more likely to suffer from low self-esteem, anxiety, depression and other loss of dignity (Dong et al., 2021; Zhou et al., 2022). In severe cases, the recovery process of the disease is delayed, patient satisfaction with life is reduced, and the desire to accelerate the death of the patient and the emergence of suicidal thoughts and other negative emotions can even occur. It is important to pay attention to their dignity level.

Meaning of life is an important indicator of quality of life and mental health (Costin and Lignoles, 2020), the stronger the meaning of life is, the better the ability to adjust to major changes and stressful events in life, and the better the quality of life is (Heintzelman et al., 2013). Teques et al. (2016) pointed out that improving one’s meaning of life can enhance their subjective sense of well-being, improve their satisfaction with life, alleviate the psychological burden and the risk of suicide, and alleviate other negative psychological states, promote healthy development of the body and mind and improve quality of life.

Self-perceived burden refers to the psychological feeling that patients are more dependent on their family members in terms of care, emotion, and finance due to their own illness and care needs, which results in dragging down their family members and becoming a burden to the family (Yeung et al., 2019). Luo et al. (2021) noted that a high level of self-perceived burden can lead to a decline in the quality of life of patients and the emergence of suicidal tendencies and behaviors. Reducing the burden of self-perception can help increase patients’ confidence in coping, enhance their compliance behavior, improve their self-care ability and quality of life, and increase their level of hope and dignity. Existing studies have focused on the current situation and influencing factors of dignity, meaning in life, and self-perceived burden, but few studies have investigated the mediating relationships among these three factors (McIlfatrick et al., 2017; Oechsle et al., 2014; Wang et al., 2019).

Chochinov’s theoretical model of dignity suggests that there is an internal link between disease-related problems, factors that maintain individual dignity, and the dignity of society (Chochinov et al., 2002b). Based on this dignity theory model, this study takes the meaning of life as a factor of maintaining individual dignity and the self-perceived burden as a factor of social dignity, considers the influence mechanism of the self-perceived burden as a mediator between the meaning of life and the level of dignity in patients with disease-related problems, and then provides a theoretical basis for preventing a lack of dignity in patients with disability.

## 2 Methods

### 2.1 Participants

A total of 229 patients with incapacity from July to October 2022 in Sichuan Province, China were selected for the study. The required sample size of the study was 5–10 times the total number of entries (Wang, 2009). There were 45 entries in this study, the required sample size was 225 ~ 450 cases, and the finalized sample size was 230 cases. All the research subjects voluntarily participated in this study and signed the consent form. The study was approved by the University Ethics Committee (2023No. 110).

The inclusion criteria were as follows: (1) disability and semidisability as measured by basic activities of daily living (BADL); (2) age ≥ 18 years; and (3) ability to read and understand words.

The exclusion criteria were as follows: (1) mental illness and (2) cognitive impairment.

### 2.2 Measures

#### 2.2.1 General information questionnaire

A general information questionnaire included gender, age, mode of residence, literacy, marital status, household income, mode of payment, duration of illness, caregiver identity, and comorbidities.

#### 2.2.2 Meaning in life questionnaire (MLQ)

The scale was first developed by Steger et al. (2006). This study used the Chinese version of the scale (Wang, 2013), with a total of 10 entries. It includes 2 dimensions of having meaning and seeking meaning, where entries 2, 4, 7, 8, and 9 are having meaning dimensions and the rest are seeking meaning dimensions. Each item was scored on a 7-point Likert scale, with 1 indicating “very inconsistent,” 7 indicating “very consistent,” and item 2 reverse scored, with higher scores indicating greater meaning in life. The Cronbach’s coefficient of the scale was 0.837, with good reliability and validity.

#### 2.2.3 Self-perceived burden scale (SPBS)

This scale was developed by Cousineau et al. (2003). This study used the Chinese version of the scale (Yanyan and Jiang, 2010), which included 10 items related to the 3 dimensions of physical burden, economic burden, and emotional burden, and each item was scored on a 5-point Likert scale, with “Never,” “Occasionally,” “Sometimes,” “Often,” and “Always” scoring 1–5, respectively. The scale total is the sum of the scores of the entries (5 to 50 points), and the higher the score is, the greater the self-perceived burden. A total score of <20 was classified as no self-perceived burden, 20–29 was classified as mild self-perceived burden, 30–39 was classified as moderate self-perceived burden, and ≥ 40 was classified as severe self-perceived burden. The internal consistency reliability of the scale was 0.91, with good reliability and validity.

#### 2.2.4 Patient dignity inventory (PDI)

This scale was developed by Chochinov (2002), and the Chinese version of the scale was created by Ge et al. (2016). The scale includes 5 dimensions, symptom distress, existential distress, independence, social support, and peace of mind, with 25 entries. Each item is rated on a 5-point Likert scale, with 1 indicating “no distress” and 5 indicating “very serious distress.” The total score of the scale ranged from 0 to 125 points. A total score < 25 points indicated that there was no damage to one’s dignity, 25–50 points indicated that one’s dignity was mildly damaged, 50–75 points indicated that one’s dignity was moderately damaged, and 75–125 points indicated that one’s dignity was severely damaged. A total score > 50 points indicated that there was a significant loss of dignity, and the higher the score was, the more severe the degree of damage to dignity was. The Cronbach’s alpha coefficient and split-half reliability were 0.924 and 0.885, respectively, with good reliability and validity.

### 2.3 Data collection

This study used both electronic and paper questionnaires to collect data, and the data collectors were uniformly trained by the members of the team before collection. The header of the questionnaire explains in detail the purpose and significance of the study, the research methodology, etc., and emphasizes that the data are strictly confidential and for research use only. (1) Electronic questionnaire: The QR code of the electronic questionnaire was generated by the Questionnaire Star platform. The research subjects scanned the QR code and read the preface (≥10 s) and then clicked “Confirm to participate in the study” to enter the questionnaire interface. If they chose to “do not participate in the study,” the questionnaire was closed. (2) Paper questionnaire: The data collector used a unified, standardized guide to explain the purpose and significance of the study to the research subjects. The research subjects agreed to participate in the study, signed the informed consent form, and then answered the questionnaire on the spot. If there were any questions, the guide was used to explain the questionnaire, avoiding the use of guiding language, and after the answer was completed, the data collector checked and recovered the questionnaire.

### 2.4 Quality control

The experts were included in the discussion before the start of the project to fully justify the scientific validity and feasibility of the research design and the questionnaire. Research subjects were identified in strict accordance with the inclusion and exclusion criteria during the survey. Clinical nursing staff with theoretical knowledge and operational skills provided patients with disease guidance and health education knowledge and collected survey data after establishing good nurse–patient relationships, thus ensuring the authenticity and validity of the information. To ensure the quality of the questionnaire, the following exclusion criteria were used: (1) The missing values of the questionnaire were > 3 (the median or mean value was used to fill in within 3). (2) With the same cell phone, the computer/IP can only answer once, and multiple answers to the first answer prevail. (3) Answering time < 100 s was considered an invalid questionnaire. At the end of the survey, the person in charge of the subject eliminated the invalid questionnaires, double-checked the data and entered them, and the statistical professionals instructed the data analysis methods of this study.

### 2.5 Data analysis

Microsoft Excel 2010 was used to organize the data, and IBM SPSS 27.0 and IBM AMOS 24.0 were used for data analysis. Measurement data that conformed to the positive-tai distribution are expressed as the mean ± standard deviation, and counting information is expressed as the frequency and percentage. The correlations among self-perceived burden, meaning in life, and dignity level of the disabled patients were analyzed by Pearson correlation analysis; the mediating role of self-perceived burden between meaning in life and dignity level of the disabled patients was analyzed by constructing a structural equation model through AMOS 24.0; and the significance of the mediating effect was tested by using a bias-corrected percentile bootstrap. *p* < 0.05 was considered to indicate a statistically significant difference.

## 3 Results

### 3.1 General information of the study subjects

A total of 230 questionnaires were distributed in this study, and 229 valid questionnaires were recovered, for a recovery rate of 99.57%. Detailed general information on the 229 study participants, comprising 77 males (33.6%) and 152 females (66.4%), is shown in Table 1.

**Table 1:** General information of the study subjects (*n* = 229).

		Number of cases	Percentage
Sex	Male	77	33.6
	Female	152	66.4
Age	18–35	117	51.1
	35–65	43	18.8
	≥65	69	30.1
Residency	Living alone	42	18.3
	Living with family	187	81.7
Education	Junior high school and below	67	29.3
	High school and junior college	47	20.5
	College and above	115	50.2
Marital status	Unmarried	113	49.3
	Divorced	11	4.8
	Bereaved	31	13.5
	Married	74	32.3
Incomes	≤2,000	67	29.3
	2,000 ~ 4,000	68	29.7
	4,000 ~ 6,000	43	18.8
	≥6,000	51	22.3
Payment method	Cooperative medical care	46	20.1
	Social health insurance	121	52.8
	Self-financed	62	27.1
Duration of illness	<1	146	63.8
	1 ~ 2	42	18.3
	3 ~ 5	21	9.2
	≥5	20	8.7
Caregiver	Spouse	75	32.8
	Sons and daughters	99	43.2
	Siblings	47	20.5
	Parents	83	36.2
	Grandparents	16	7.0
	Grandchildren	11	4.8
	Other	14	6.1
Complication	Hypertension	70	30.6
	Diabetes	46	20.1
	Hyperlipidemia	21	9.2
	Coronary heart disease	18	7.9
	Other	15	6.6

### 3.2 Current status of meaning of life, self-perceived burden and level of dignity

The results of the study showed that disabled patients had scores of 46.27 ± 9.71 for meaning in life, 23.55 ± 9.83 for self-perceived burden, and 43.38 ± 22.04 for level of dignity, and the scores of the dimensions are detailed in Table 2.

**Table 2:** Current status of meaning of life, self-perceived burden and level of dignity.

Scale	Dimension	Score	Mean value of entries
Meaning of life	Totals	46.27 ± 9.71	5.14 ± 1.50
	Having meaning	24.77 ± 5.65	4.95 ± 1.55
	Seeking meaning	21.50 ± 4.85	5.38 ± 1.44
Self-perceived burden	Totals	23.55 ± 9.83	2.36 ± 1.13
	Physical burden	4.40 ± 1.94	2.20 ± 1.07
	Economic burden	4.93 ± 2.19	2.47 ± 1.17
	Emotional burden	14.22 ± 6.08	2.37 ± 1.14
Dignity level	Totals	43.38 ± 22.04	1.74 ± 1.02
	Symptomatic distress	12.65 ± 6.42	1.81 ± 1.04
	Existential distress	5.31 ± 2.88	1.77 ± 1.05
	Independent	9.96 ± 5.25	1.66 ± 1.00
	Social support	5.13 ± 2.80	1.71 ± 1.01
	Peace of mind	10.33 ± 5.45	1.72 ± 1.01

### 3.3 Correlations between meaning of life, self-perceived burden and level of dignity

Pearson correlation analysis revealed that meaning of life was negatively correlated with self-perceived burden (*r* = −0.325, *p* < 0.001), meaning of life was negatively correlated with dignity (*r* = −0.526, *p* < 0.001), and self-perceived burden was positively correlated with dignity (*r* = 0.460, *p* < 0.001), as detailed in Table 3.

**Table 3:** Correlations between meaning of life, self-perceived burden and level of dignity.

	MLQ	Seeking meaning	Having meaning	SPBS	Physical burden	Economic burden	Emotional burden	PDI	Symptomatic distress	Existential distress	Independent	Social support	Peace of mind
MLQ	1												
Seeking meaning	0.912**	1											
Having meaning	0.936**	0.708**	1										
SPBS	−0.325**	−0.296**	−0.303**	1									
Physical burden	−0.263**	−0.237**	−0.248**	0.918**	1								
Economic burden	−0.333**	−0.315**	−0.302**	0.935**	0.830**	1							
Emotional burden	−0.321**	−0.290**	−0.302**	0.987**	0.867**	0.886**	1						
PDI	−0.526**	−0.433**	−0.532**	0.460**	0.402**	0.424**	0.463**	1					
Symptomatic distress	−0.530**	−0.446**	−0.528**	0.480**	0.417**	0.444**	0.482**	0.977**	1				
Existential distress	−0.503**	−0.405**	−0.517**	0.448**	0.405**	0.412**	0.446**	0.955**	0.912**	1			
Independent	−0.478**	−0.391**	−0.486**	0.411**	0.349**	0.374**	0.419**	0.965**	0.924**	0.896**	1		
Social support	−0.484**	−0.400**	−0.489**	0.389**	0.338**	0.354**	0.394**	0.952**	0.919**	0.908**	0.911**	1	
Peace of mind	−0.528**	−0.431**	−0.537**	0.464**	0.412**	0.431**	0.463**	0.970**	0.931**	0.929**	0.907**	0.897**	1

*p < 0.05, **p < 0.01, ***p < 0.001.

### 3.4 The mediating role of self-perceived burden between meaning of life and level of dignity

**Figure 1:**
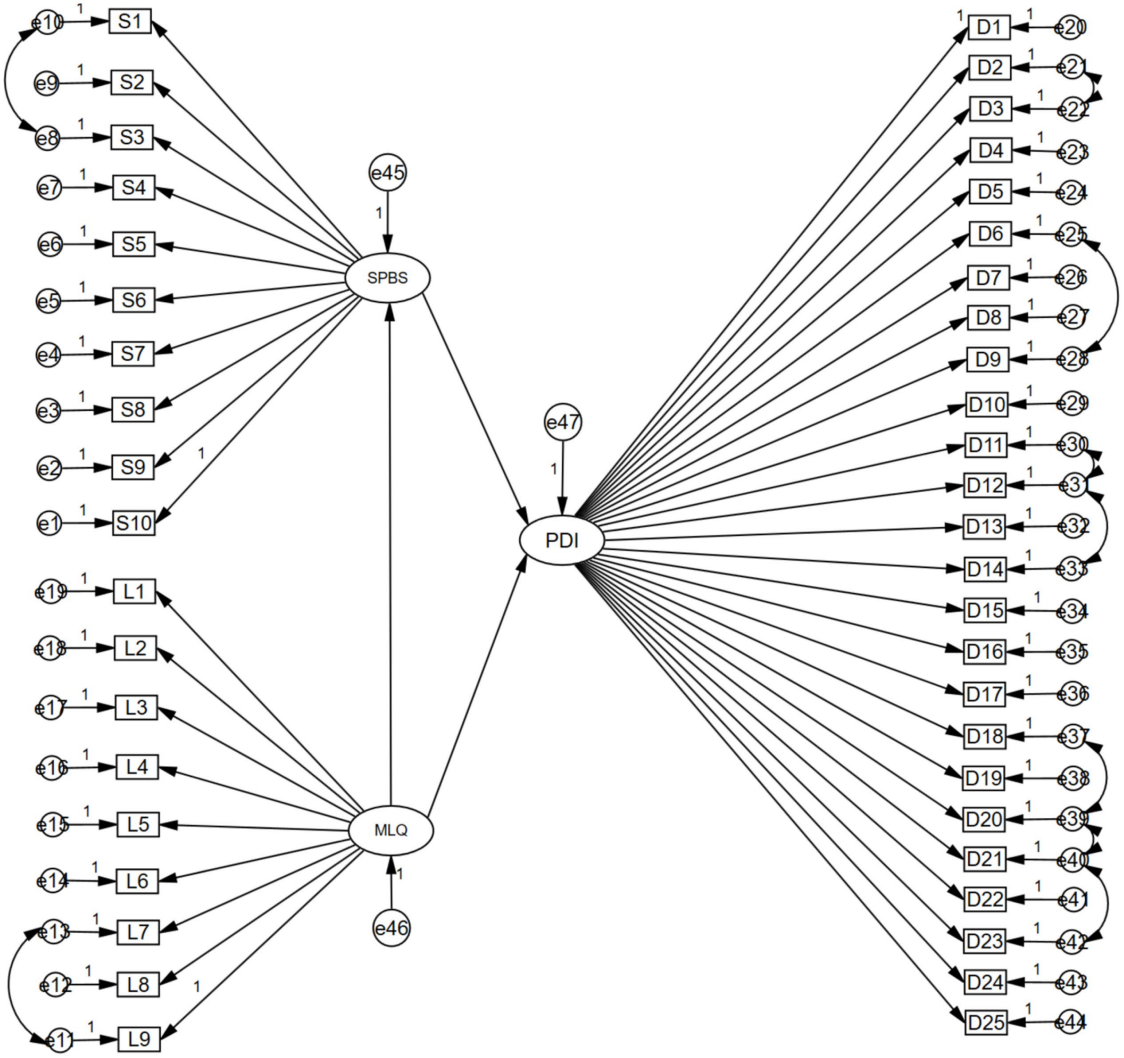
Model of the mediating effect of self-perceived burden between meaning in life and dignity in disabled patients.

To further analyze the relationship between self-perceived burden and the level of dignity and the meaning of life for disabled patients, this study used Chochinov et al.’s (2002b) dignity model as the theoretical basis, taken the meaning of life as the independent variable, the level of dignity as the dependent variable, and self-perceived burden as the mediator, and established a structural equation model combined with Amos 24.0. The final results showed that the values were within the acceptable range. The structural equation model fit well. See Table 4 for details. The standardized coefficients of each path in the model, i.e., meaning of life on self-perceived burden (*β* = −0.338, *p* < 0.001), meaning of life on level of dignity deficit (*β* = −0.409, *p* < 0.001), and self-perceived burden on level of dignity deficit (*β* = 0.340, *p* < 0.001), are all significant (Figure 1).

**Table 4:** Fit indices for structural equation modeling.

Variable	X^2^/df	GFI	CFI	NFI	IFI	RMSEA
Adaptation standards	<3	>0.90	>0.90	>0.90	>0.90	<0.10
Results	2.326	0.713	0.900	0.838	0.901	0.078

### 3.5 Significance test for mediated effects

The mediating effect test was conducted using the bias-corrected percentile bootstrap method with a set sampling number of 5,000 and a confidence interval of 95%. The final results showed that the confidence interval of the path of the mediating effect of self-perceived burden on the level of dignity through meaning in life was (LLCI = −0.4038, ULCL = −0.1042), which did not contain 0, indicating that this mediating effect was significant and that the size of the mediating effect was −0.2386. In addition, after controlling for the mediating effect of self-perceived burden, the effect of the independent variable, meaning in life, on the dependent variable, dignity, was also significant (LLCI = −1.2056, ULCL = −0.7062). Therefore, there is a mediating role of self-perceived burden on the effect of meaning of life on the level of dignity. Therefore, this model is a partial mediation model in which the indirect effect accounts for 19.98% of the total effect. See Table 5 for details.

**Table 5:** Path relationships between self-perceived burden at the level of dignity and sense of meaning in life.

Effect	Trails	Effect size	Percentage	95% CI	
				LLCI	ULCL
Direct effect	Meaning in life → Dignity	−0.9559	80.02%	−1.2056	−0.7062
Indirect effect	Meaning in life → Self-perceived burden → Dignity	−0.2386	19.98%	−0.4038	−0.1042
Aggregate effect		−1.1945	100%	−1.4471	−0.9419

## 4 Discussion

### 4.1 Current status of disabled patients’ meaning of life, self-perceived burden and dignity

Enhancing the meaning of life can help patients think about and explore their own value of life, trigger positive stress coping styles, promote harmony in interpersonal relationships, and enhance their sense of well-being and quality of life. The results of this study showed that the largest proportion of disabled patients had an unclear meaning of life, which is similar to the results of the study conducted by Xie et al. (2022), indicating that there is a great deal of room for improvement in the meaning of life of disabled patients. The scores of the dimensions showed that the scores of the dimension of seeking meaning in life were greater than those of having meaning in life, similar to the results of the study conducted by Costanza et al. (2020). Alter and Hershfield (2014) noted that patients’ seeking meaning in life was dominant in the early stage of their lives and that their experience of meaning in life increased with age and then shifted gradually from the state of seeking to the state of possessing meaning in their middle-aged and old-aged lives. In this study, 51.1% of the patients were 18–35 years old, were young and were in the early stage of life; therefore, the patients in this study scored higher in seeking meaning in life.

A low level of self-perceived burden is conducive to enhancing the self-confidence of sick individuals in restoring their health, improving their ability to take care of themselves, and improving their self-management behaviors (Kowal et al., 2012). In this study, the proportion of people with self-perceived burdens was 67.69%, and the average score was 23.55 ± 9.83, which indicated that the phenomenon of self-perceived burdens was common in patients with incapacitation and that its severity was mild, similar to the results of Ditchman et al.’s (2017) study. The dimensions showed that patients’ financial burden was greater, followed by their emotional burden, and their physical burden was lower. Wei et al. (2020) noted that the physical burden of patients with incapacity was greater, followed by economic burden, and emotional burden was lower, which was different from the findings of the present study. The reason for this is that the patients in this study were hospitalized patients who were undergoing medical treatment, and most of them were in the acute phase of the disease, at which time the economic burden of the patients was in a heavier state, while their physical burden was alleviated by medical care measures, so the patients’ economic burden was the heaviest, and their physical burden was lighter.

A serious loss of dignity may lead to negative psychology, such as isolation, sadness, helplessness, etc., affecting mental health, and in serious cases, it may lead to adverse consequences, such as patients’ desire for early or accelerated death. The results of this study revealed that 64.19, 27.08 and 8.73% of the disabled patients experienced mild, moderate and severe dignity impairment, respectively, which indicates that most disabled patients generally experience dignity impairment, and most of these patients exhibit mild dignity impairment, which is similar to the results of Henry et al.’s (2014) study. Dong et al. (2021) noted that disabled patients have a lower level of dignity due to the difficulty of self-care and the long-term experience of physical disease. Therefore, there is a need to focus on psychological status, improving social support, and improving the level of dignity while maintaining the patient’s functioning.

### 4.2 Correlations among meaning of life, self-perceived burden and dignity among disabled patients

The basic psychological needs satisfaction model of the meaning of life indicates that the satisfaction of psychological needs is the fundamental motivation for individuals to obtain meaning in life (Kim and Beehr, 2020). The results of this study show that the meaning of life of disabled patients is negatively correlated with self-perceived burden, indicating that the clearer the meaning of life of disabled patients is, the lighter the self-perceived burden, which is in line with the results of Yıldırım and Arslan (2021). That pointed out that patients with a clear meaning of life have a positive view of life, and when patients face stress or burden due to a decline in physical and social functioning and difficulty fulfilling life needs. Those with a clear meaning of life are able to activate their own resilience, reexamine and adjust harmful beliefs, and transform burden into positive cognition, thus reducing their perception of stress, which is to some extent conducive to the reduction of the self-perceived burden. Therefore, to reduce the self-perceived burden of patients with disability, we should take controlling the development of the disease as the current goal of life while actively cooperating with the treatment to improve their meaning of life.

McPherson et al. (2010) reported a significant correlation between self-perceived burden and patients’ depression, anxiety, loss of dignity, and despair. The results show that the self-perceived burden and the level of loss of dignity in patients with incapacity are positively correlated, i.e., the greater the self-perceived burden is, the more serious the level of loss of dignity, which is similar to the results of Bagheri et al.’s (2018) study. Chochinov et al. (2002a) pointed out that patients who are incapable of changing from being a caregiver to being cared for are forced to withdraw from social work and have difficulty taking on the responsibility of their family, and the conflict of roles and behaviors aggravates their self-perceived burden. Along with the increase in negative emotions, the sense of social value decreases, patients gradually feel disrespected, and the phenomenon of lack of dignity occurs. Therefore, helping patients better maintain their self-esteem should be the overall goal of treatment and care for patients with disabilities, and respect for patients’ personal autonomy should be taken as the basic value under the premise of symptomatic care to maximize patients’ sense of dignity.

Liu et al. (2021) noted that the use of positive means to improve the meaning of life in advanced cancer patients can effectively prevent them from experiencing a loss of dignity, and there is a strong relationship between the two. The results of this study show that meaning of life of disabled patients is negatively correlated with the level of dignity loss. i.e., the clearer the meaning of life of disabled patients is, the lower their sense of dignity loss is. Studies have pointed out that a meaning of life not only helps patients to effectively control their disease but also helps to realize personal happiness and gain a sense of value (Guerrero-Torrelles et al., 2017), thus enhancing the level of dignity, which is consistent with the results of this study. Studies have pointed out that dignity therapy can effectively enhance patients’ sense of dignity and meaning of life (Hack et al., 2010; Cui et al., 2023), reduce patients’ inner pain, and increase their desire to live to make them physically and mentally comfortable and improve their quality of life as a whole. However, most of the studies on dignity-preserving therapy have focused on patients with advanced cancer, and the effect on patients with incapacitation needs to be further explored.

### 4.3 Mediating effects of self-perceived burden among disabled patients’ meaning in life on their dignity level

Van Gennip et al.’s (2013) Dignity Model of Illness argues that illness itself does not lead to a decrease in the sense of self-worth but rather acts on the patient’s individual self by triggering physical and psychological changes, such as his or her sense of significance, which ultimately leads to the patient’s level of dignity. The results of this study show that the meaning of life in patients with disability has a negative predictive effect on the level of dignity and that the meaning of life has an indirect effect on the level of dignity through the burden of self-feeling, i.e., the self-perceived burden plays a partially intermediary role between the meaning of life and the level of dignity, indicating that meaning of life of patients with disability is able to affect the level of the self-perceived burden, which in turn contributes to the change in the level of the dignity deficit, which is in line with Gennip’s dignity model theory.

McPherson pointed out that disabled patients, in the face of limited motor function, daily self-care ability to decline, may need help bathe, need to assist in defecation and other problems; at this time, the patient will experience anxiety, depression and other self-perceived burden phenomena, and self-perceived burden and the patient’s sense of dignity are highly correlated with the fracture. This burden is likely to lead to the patient experiencing sadness, despair, and isolation emotions. In severe cases, patients may even experience a loss of dignity, such as a desire to hasten death. Patients with a high meaning of life tend to have a positive view of life, and when their self-care ability decreases and their participation in social activities decreases, the positive meaning of life of such patients often serves as a protective buffer to help them actively cope with adverse psychological emotions, improve their tolerance of physical symptoms, and maintain their self-perceived burden at a lower level (Weru et al., 2020). A low level of self-perceived burden can help patients better adapt to role-behavior conflicts, reducing anxiety, depression, despair and other adverse emotions, enhancing their subjective sense of well-being, and avoid patient dignity deficit (Rejnö et al., 2020). Therefore, this mediating effect can be explained and is consistent with the conclusions of Wei et al.’s (2020) study, both emphasizing the connectivity of self-perceived burden between meaning in life and the level of dignity deficit. Therefore, healthcare professionals not only need to pay attention to changes in the condition of disabled patients but also need to strengthen their psychological care and comfort and help patients establish a positive meaning in life to improve the level of self-perceived burden, prevent patients from experiencing the phenomenon of dignity deficit, and satisfy their needs and higher levels of psychological needs.

### 4.4 Limitations

Structural equation modeling revealed that self-perceived burden partially mediated the relationship between meaning in life and the level of dignity of patients with incapacitation. There are two limitations: (1) The study subjects were not differentiated by the degree of incapacity, and it was not determined whether the patients were in temporary or long-term incapacity, which may have affected the results of the study to a certain extent, and the study subjects were further refined in a later study. (2) This was a single-center cross-sectional study, the sample size was small, there may be research bias, and subsequent large-sample, multicenter investigative research.

## 5 Conclusion

This study used the mediation effect model to confirm the partial mediation effect of self-perceived burden between the meaning of life and the dignity level of patients with incapacitation, indicating that the meaning of life of patients with incapacitation can affect the level of self-perceived burden, which in turn promotes changes in the level of their dignity deficit. Therefore, on the basis of paying attention to the patient’s condition, disabled caregivers need to fully understand the patient’s psychological demands and take targeted interventions to help the patient establish a positive meaning of life so that the patient can cope with the disease in a positive state of mind and prevent the patient from experiencing an increase in the self-perceived burden and a sense of loss of dignity.

## Data Availability

The original contributions presented in the study are included in the article/supplementary material, further inquiries can be directed to the corresponding author.
